# Anti-infectivity of camel polyclonal antibodies against hepatitis C virus in Huh7.5 hepatoma

**DOI:** 10.1186/1743-422X-9-201

**Published:** 2012-09-16

**Authors:** Esmail M EL-Fakharany, Nawal Abedelbaky, Bakry M Haroun, Lourdes Sánchez, Nezar A Redwan, Elrashdy M Redwan

**Affiliations:** 1 Antibody Laboratory, Protein Research Dept., Genetic Engineering and Biotechnology Research Institute, City for Scientific Research and Technology Applications, New Borg EL-Arab, Alexandria, 21394, Egypt; 2 Botany and Microbiology Dept., Faculty of Science, Alazhar University, Cairo, Egypt; 3 Tecnología de los Alimentos, Facultad de Veterinaria, Universidad de Zaragoz, Miguel Servet 177, Zaragoza, 50013, Spain; 4 Biological Sciences Dept., Faculty of Science, King Abdulaziz University, P.O. Box 80203, Jeddah, 21589, Kingdom of Saudi Arabia

**Keywords:** Camel milk proteins, Hepatitis C virus, Screening, Infectivity

## Abstract

**Purpose:**

To extend the study of the camel milk proteins which have antiviral activity against HCV, camel naïve polyclonal IgGs, α-lactalbumin were purified from camel milk and their anti-HCV effect was examined using PBMCs and Huh7.5 cell-lines. They were compared with the activity of human polyclonal IgGs and camel lactoferrin and casein.

**Material and methods:**

Three types of experiments were performed on PBMCs and HuH7.5 cell. HCV was directly incubated with the purified proteins and then mixed with both cell types, or the proteins were incubated with the cells and then exposed to HCV, or the HCV pre-infected cells were treated with the proteins to inhibit intracellular replication. The proteins were added to cells or virus at different concentrations and time intervals.

**Results:**

Pretreated PBMCs and Huh7.5 cells with milk proteins were not protected when exposed to HCV infection. The direct interaction between HCV and camel IgGs and camel lactoferrin (cLf) led to a complete inhibition of HCV entry into cells, while casein, α-lactalbumin and human IgGs failed to inhibit HCV entry at any tested concentration. Camel IgGs showed ability to recognize HCV peptides with a significant titer (12 × 10^3^) in comparison with human IgGs which failed to do it. Camel lactoferrin was capable of inhibiting the intracellular HCV replication at concentrations of 0.25-1.25 mg/ml.

**Conclusion:**

Camel milk naïve polyclonal IgGs isolated from camel milk could inhibit the HCV infectivity and demonstrated strong signal against its synthetic peptides. Lactoferrin inhibit the HCV infectivity started from 0.25 mg/ml. However, α-lactalbumin, human IgGs and casein failed to demonstrate any activity against HCV infectivity.

## Background

Hepatitis C is a global health problem and represents a major cause of liver disease and a socioeconomic burden [1], without the existence of any protective vaccine or effective drug. The potential treatment of the recognized effective drugs (interferon plus ribavirin and/or pegylated interferon) for hepatitis C virus (HCV) lead to an impressive sustained virological response that did not exceed 56 % [1].

Camel milk is often used as an adjuvant treatment for several chronic diseases, such as diabetes mellitus, or in allergic patients [2-7]. However, scientific basis for the positive effect of camel milk improving the health status of those patients (especially those suffering from hepatitis has to be better understood. Camel milk possesses a protein system constituted by two major classes of proteins: caseins and whey proteins. Caseins account for 80 % (w/w) of the total milk protein content [8] and whey contains numerous proteins such as immunoglobulins, α-lactalbumin, lactoperoxidase, lysozyme and lactoferrin, among other proteins with biological functions [9]. Lactoferrin plays an important and multifunctional role in innate and specific host defense against infection by microorganisms, alone or with other milk proteins such as lysozyme and immunoglobulins, [10-13]. Lactoperoxidase is present in the milk of many species and catalyzes the oxidation of some organic and inorganic substrates by hydrogen peroxide, such as thiocyanate in milk, producing derivate compounds with antibacterial activity [14]. Lysozyme is another antibacterial protein present in milk, tears, saliva, and other secretions of mammals. The antibacterial activity of lysozyme is exerted by damaging the cell wall of some bacteria. It has been shown a great activity of camel milk lysozyme against *Salmonella typhimurium* compared to other types of lysozyme [9].

Camels have a unique and special class of antibodies which were referred to as Heavy-chain antibodies (HCAbs) because they lack the classical light-chain and are composed of a homodimer of heavy-chains [15]. α-lactalbumin is a low molecular weight acidic protein (14.2 KDa) present in the whey fraction of milk. Recently, the capacity of defending the newborn from pathogenic microorganisms has been putatively ascribed to α-lactalbumin [16-18]. α-lactalbumin itself does not possess any antimicrobial activity; however, when it changes to a particular conformation it acquires antimicrobial and antitumoral properties [16]. The aim of this work was to study: the camel naive polyclonal IgGs and α-lactalbumin inhibitory activity against HCV, compare their inhibitory activity with human polyclonal IgGs, confirm and compare with the previously published results of camel milk proteins (lactoferrin, casein) activity on HCV entry and replication in PBMCs and Huh7.5 cells, inaddition to their positive control roll.

## Results

### Antibody reactivity against HCV peptides

**Figure 1:**
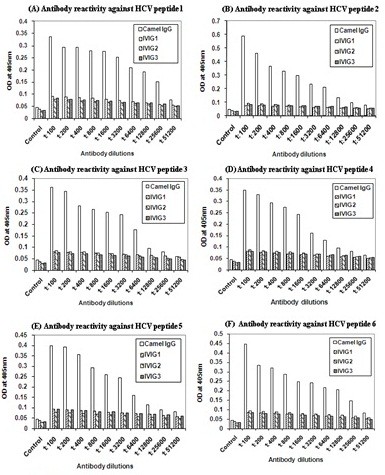
Reactivity of camel IgGs and human IVIG-1, IVIG-2 and IVIG-3 against HCV synthetic peptides 1–6 (A-F), in serial dilutions.

The HCV peptides were designed to be mostly conserved within the core and envelope of HCV genotypes. The tested human polyclonal IgGs presented values of reactivity against HCV-synthesized peptides similar to that of the control, though camel antibodies gave positive results as is shown in Figure 1. Camel naïve polyclonal IgGs significantly recognized all the types of HCV peptides with an affinity up to 25 × 10^3^. Both core peptide 2 and envelope 2 peptide 6 released stronger signal than the rest of peptides. However, human polyclonal IVIGs showed very low reactivity with any of the HCV synthesized peptides (Figure 1).

### Cytotoxic effect of camel IgGs, α-lactalbumin, Lf, casein, or IVIGs

We tested the cytotoxic effects of camel IgGs, α-lactalbumin, cLf, casein and IVIGs on PBMCs and Huh7.5 cells to exclude the possibility of HCV elimination caused by reduced cell viability. We also wanted to investigate the *in vitro* side effects of those proteins on the two types of cells (normal and hepatoma cells). Cell viability determined by MTT method was compared with the untreated PBMCs and Huh7.5 cells and the results are shown in Table 1. Our results indicated that camel or human proteins tested at 1.0 or 2.0 mg/ml did not have any effect on the viability of the PBMCs or any cytotoxic effect after 4 days of incubation period, as it presented values above 89 %. Similar results were obtained when using Huh7.5 cells, unless for the incubation with camel casein that reduced cell viability to 10 % and 5 % at protein concentrations of 1.0 and 2.0 mg/ml, respectively.

**Table 1:** Cell viability of Huh7.5 (A) and PBMC (B) incubated with different proteins determined by MTT method

**Protein**	**Protein concentration (A)**	**Protein concentration (B)**		
Concentration	1.0 mg/ml	2.0 mg/ml	1.0 mg/ml	2.0 mg/ml
Control	100	100	100	100
cLf	90	86	100	100
Camel casein	10	5	100	99
Camel IgGs	86	85	100	100
Camel α-lactalbumin	87	84	100	100
IVIG-1	87	85	100	98
IVIG-2	86	84	100	99
IVIG-3	87	85	100	100

The values are expressed as percentage of control cell viability.

### Neutralization and protection effects of camel IgGs, α-lactalbumin, cLf, casein and human IVIGs

**Figure 2:**
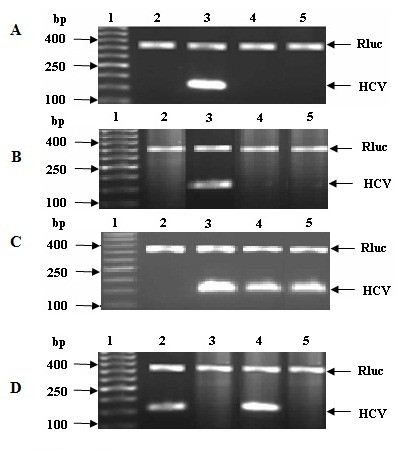
**Activity of cLf (A), camel IgGs (B) and camel α-lactalbumin (C) against HCV on Huh7.5 cells.** In A, B and C, lanes 1, 2, 3 are DNA ladder, negative and positive control, respectively; in D, lanes 1, 2, 3 are DNA ladder, positive and negative control, respectively. Lanes 4 and 5 are cLf, IgGs or α-lactalbumin through direct interaction with HCV at concentrations of 0.5 and 1.0 mg/ml, respectively. The activity of camel casein at a concentration of 1.0 mg/ml (**D**) against HCV through direct interaction on PBMCs and Huh7.5 is shown in lanes 4 and 5.

**Figure 3:**
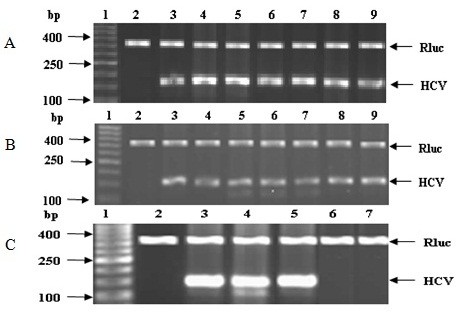
**Activity of human IVIGs against HCV entry into Huh7.5, tested through direct interaction between viral particles and proteins.** All panels contain DNA ladder (lane 1), negative control (lane 2) and amplified 174 bp of HCV in positive control (lane 3). (**A**) Lanes 4–9 show the direct interaction between HCV and IVIG-1, IVIG-2, and IVIG-3 at concentrations 0.5 and 1.0 mg/ml, respectively. (**B**). Lanes 4–9 represent cLf, IgGs, lactalbumin, human IVIG-1, IVIG-2 IVIG-3 at concentrations of 1.0 mg/ml, respectively. (**C**) Activity of camel casein against HCV entry into PBMCs and Huh7.5 cells. Infected PBMCs incubated with casein at concentrations of 0.5 and 1.0 mg/ml (lanes 4 and 5) and Huh7.5 cells incubated with casein at concentrations of 0.5 and 1.0 mg/ml (lanes 6 and 7).

Camel IgGs and lactoferrin at concentrations of 0.5 and 1.0 mg/ml were able to completely inhibit or prevent the entry of HCV particles into PBMCs and Huh7.5 cells as shown in Figure 2. However, camel *α-*lactalbumin and all types of IVIGs at concentrations of 0.5 and 1.0 mg/ml failed to neutralize HCV particles and prevent these particles from entry into PBMCs and Huh7.5 as shown in Figures 2 and 3. The purified camel casein could not be able either to block the HCV entry to PBMCs at concentrations of 0.5 and 1.0 mg/ml, as shown in Figure 3, though we did not detect HCV band at 174 bp in agarose gel for both concentrations of casein in Huh7.5 cells. Our results indicated that all proteins used in the assays failed to protect human PBMCs and Huh7.5 cells or block the cell receptors from HCV entry (Figure 3). It was observed that the HCV band at 174 bp was not detected in agarose gel when camel casein was added to Huh7.5 cells as it was also shown in Figure 2, which may be due to the elimination of HCV caused by a reduced viability of Huh7.5 cells as described above.

### Effect of camel IgGs, Lf, casein, α-lactalbumin and human IVIGs on replication of HCV

**Figure 4:**
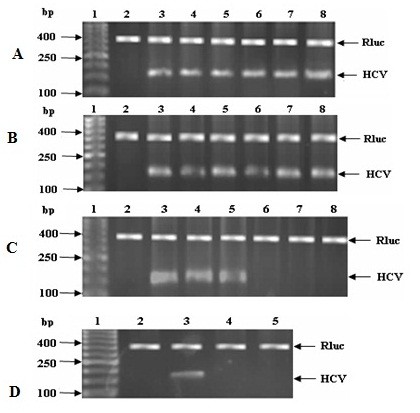
**Intracellular inhibition activities of camel IgG (A) and α-lactalbumin (B) on HCV replication in Huh7.5 cell line.** DNA ladder (lane 1), negative control (lane 2), positive control (lane 3) and lanes 4–8 show the effect of IgGs and α-lactalbumin at different concentrations (0.25, 0.5, 0.75, 1.0 and 1.25 mg/ml) on HCV-infected Huh7.5 cells after four days of treatment. (**C**) Lanes 4–8 show the effect of first dose of cLf at various concentrations (0.25, 0.5, 0.75, 1.0 and 1.25 mg/ml) on HCV-infected Huh7.5 cells after first dose of treatment. (**D**) Lanes 4–5 show the effect of cLf (0.25 and 0.5 mg/ml) on HCV-infected Huh7.5 after 8 days.

Camel lactoferrin, casein, IgGs, *α-*lactalbumin and IVIGs at concentrations of 0.25, 0.5, 0.75, 1.0 and 1.25 mg/ml were tested for their *in vitro* ability to inhibit or prevent HCV particles replication inside the infected PBMC and Huh7.5 cells. Camel lactoferrin could inhibit HCV replication only at concentrations from 0.75 to 1.25 mg/ml after 96 h and at concentrations from 0.25 mg/ml after a second dose of treatment (another 4 days) as shown in Figure 4. However, camel IgGs, *α*-lactalbumin, casein and IVIGs failed to prevent HCV replication at any concentration used (0.25-1.25 mg/ml) after 4 days of treatment as shown in Figure 4. However, HCV band at 174 bp was not detected when we treated HCV infected Huh7.5 cells with camel casein at any concentration (Figure 4), that elimination of HCV was caused again by a reduced viability of Huh7.5 cells, as it has been described above.

## Discussion

Nowadays, many research lines are directed to study some components derived from natural sources, which might simply improve our life by protecting us and improving our health. Historically, camel’s milk has been used for medical purposes and treatment of some diseases. Many useful properties are assigned to camel’s milk that has been traditionally used for the treatment of tuberculosis, gastroenteritis and allergy, and is also drunk as a tonic. The beneficial properties of camel’s milk, among them its antimicrobial activity, can be attributed to substances such as proteins, lipids, carbohydrates and vitamins. Among milk proteins, lactoferrin, IgGs, α-lactalbumin, casein, lactoperoxidase, lysozyme, and some peptides are the main components that have been suggested to possess those activities [19-22].

In this work, we studied the antiviral activity of camel IgGs, α-lactalbumin, lactoferrin, and casein in comparison with three human commercial intravenous immunoglobulins G, against hepatitis C virus by determining its infectivity in Huh 7.5 and PBMCs cell lines. The results showed that camel IgGs and lactoferrin prevented HCV from entry into PBMCs and Huh7.5 cells when assessed by two *in vitro* assays. The second was an assay based on the direct interaction of proteins with the virus particles, and the first one evaluated the protection of the cells by the proteins. We observed that camel α-lactalbumin, casein and human IVIGs at concentrations of 0.5 and 1.0 mg/ml failed to inhibit HCV molecules from entry into PBMCs and Huh7.5 cells, as shown by both types of assays. However, the results obtained by electrophoretic analysis showed no band corresponding to HCV when the activity of camel casein was evaluated on Huh7.5 cells, though that was caused by a reduced viability of the cells because camel casein induced apoptosis. The viability of Huh7.5 cells was greatly reduced and consequently, the HCV amplified 174 bp band could not be detected, which confirm the previous report [23]. The apoptosis caused by camel casein is in accordance with the results published in the study of Håkansson et al. [24] in which it was reported that human casein (α-lactalbumin bound to casein was implicated in its apoptotic activity) and bovine α-lactalbumin unfolded and forming a complex with oleic acid called “human α-lactalbumin made lethal to tumor cells” (HAMLET), induced differential and significant apoptosis in several cancer lines [25,26].

We also described in a previous study [23] that camel casein at the concentrations used in the present work, initiated apoptosis in HepG2 cells, reducing their viability and consequently, the detection of HCV RNA. No antiviral activity against different virus has been either shown by intact bovine casein [27], however, these authors reported that bovine casein when was chemically modified could acquire antiviral activities [27].

The results of anti-HCV activity of cLf were in agreement with a previous study [28,29] which used cLf to inhibit HCV (genotype 4) entry into human PBMCs and with another study [30] which showed that cLf inhibited HCV entry into HepG2 cells. This antiviral activity of cLf against HCV also agree with previous studies in other cell lines that reported that human and bovine lactoferrin inhibited HCV (genotype1) entry into the non-neoplasic human hepatocyte cell line PH5CH8 [31] at a concentration limit of 2 mg/ml. Our results showed that cLf at concentrations of 0.5, 1.0 and 1.25 mg/ml inhibited HCV replication inside infected cells after the first dose of treatment (four days) and at concentrations of 0.25 and 0.5 mg/ml after the second dose of treatment (another four days).

The inhibitory activity of purified camel polyclonal IgGs against HCV found in the present work is in agreement with results reported of Martin et al. [32] who described the inhibitory activity of recombinant camel VH domain antibody biopanned against HCV NS3 protease and partially ascribed by a recent study [33]. Camel’s IgG are not limited to one major subclass IgG1, but includes three main subclasses (IgG1, IgG2 and IgG3) [32]. IgG2 and IgG3 subclasses are devoid of light chains and have heavy chains of 45 and 42 kDa, respectively [15]. It has been suggested that the functional domain (the N-terminal variable region of the heavy chain antibodies referred as VH that is the minimal intact antigen-binding fragment that can be generated) of the heavy-chain antibodies interfere with several biological processes and might make good candidates for human therapy [34]. IgG2 and IgG3 act as true competitive inhibitors by penetrating into active sites of some enzymes [32,33]. They might have inhibitory activity on human immunodeficiency viruses type 1 (HIV-1) reverse transcriptase, protease, and integrase enzymes that are crucial to the HIV-1 life cycle [34].

The potential activity of IVIG preparations was used in the treatment and prophylaxis of infectious diseases and toxin, and they were also used as specific antibodies against causative bacterial LPS [35], but at least from the information we have available there is no previous study using human IVIGs in the neutralization of hepatitis C virus *in vitro*. We found that cLf acts as a strong anti-HCV agent and camel IgG acts as an intermediate agent, while camel casein, α-lactalbumin and IVIGs failed to inhibit HCV. Camel casein was not able to prevent or neutralize HCV from cell-entry at both levels, by direct or indirect interactions. Passive immunization with antibodies for the prevention and treatment of diseases become part of medical therapy, intravenous immunoglobulin being most important plasma natural product available for therapeutic use [36-38]. One gram of intravenous immunoglobulin G (IVIG) contains 4 × 10^18^ molecules with more than 10^7^ antibody specificity. This fact leads to a tremendous increase in IVIG availability and its subsequent use in the clinical setting.

Several evidences demonstrate that neutralizing antibodies are present in patients with chronic hepatitis C, and the epitopes located within the E2 protein are important for HCV neutralization. Thus the experimental preparation made from anti-HCV-positive plasma (HCIGIV) prevented HCV infection when mixed with a virus inoculum *ex vivo* before infusion into chimpanzees. Unfortunately, the *in vivo* efficacy of HCIGIV in both chimpanzees and humans has been disappointing [39]. However, the human blood and/or plasma fractions industry exclude any blood or plasma fraction containing HCV antibodies [39]. This might explain the historical evidence that normal immunoglobulins manufactured before the screening of blood or plasma donor for HCV infection protected against hepatitis C [35,38].

## Conclusion

The importance of camel milk and its components are increasing day after day. This milk is used as a main source of nutrients in several arid and non-arid areas worldwide. Camel IgGs inhibited HCV infectivity as well as differentially recognized the envelope and core synthesised HCV peptides. Meanwhile, lactoferrin was able to block the HCV cell-entry and aborted its intracellular multiplication. Although these results are promising, more extensive works are on the run to explore the relationship between lactoferrin structure and its anti-HCV activity. In addition, deeper studies will be needed to analyze the role of native camel IgGs as anti-HCV agent.

## Materials and methods

### Protein preparation

Camel polyclonal IgGs, α-lactalbumin, lactoferrin and casein were purified from milk of 35 healthy camels as previously described [18,40]. Human polyclonal intravenous immunoglobulins (IVIG) were purchased from three companies (γ-Globulin-Korean Green Cross Cooperation (IVIG-1, KGCC), 135–989 Seoul, Korea; IVIG-2 of Octapharma, 1100 Viena, Austria; IVIG-3 of VIGAM-S BPL, Herts WD6 3BX, UK), which contain the natural ratio of IgG1, IgG2, IgG3, IgG4 without IgM or IgA (the full description of these products are available in their companies web or in reference [35].

### Cell culture and media

Huh7.5 cells were kindly donnated by Prof. Charles Rice at the Rockefeller University (New York, NY 10065–7919, USA). The culture media, cell preservation and running of the cultured cells were used as previously published [41,42]. Peripheral blood cells (PBMCs) were isolated, as reported by El-Fakharany et al. [43].

### Protein and endotoxin determination

Protein content was determined by directly measuring the absorbance at 280 nm and by the Bradford method using bovine serum albumin as standard protein [44]. The endotoxin content was checked to avoid its pyrogenic effects on the cell-culture system [45]. All purified proteins used were free of endotoxin (data not shown).

### HCV peptides design and synthesis

Harnessing the CLUSTALW multiple sequence alignments (http://www.workbench.sdsc.edu) the following sequences were extracted from Los Almos HCV sequences database http://hcv.lanl.gov. The six peptide sequences were extracted from the most conserved regions and were synthesized commercially (ANASPEC Inc., San Jose, CA 95131–1314, USA) in the following design:

Peptide-1 from the HCV core protein (DVKFPGGGQIVGGVYLLPRR)

Peptide-2 from the HCV core protein (GPRLGVRATRKTSERSQPRG)

Peptide-3 from the HCV core protein (IPKARRPEGRTWAQPGY)

Peptide-4 from the HCV core protein (IPKDRRSTGKSWGKPGY)

Peptide-5 from the HCV envelope1 protein (QHRMAWDMM)

Peptide-6 from the HCV envelope2 protein (NLQLINTNGS)

The peptides were synthesized in the amide form using the standard solid phase synthesis involving 9-flurenylmethoxy carbonyl chemistry [46] and purified by HPLC according to the producer construction.

### Infected serum samples

For all infection experiments, we utilized PCR-HCV positive serum samples of genotype 4a from an Egyptian patients (after approval by ethical committee of the Genetic Engineering and Biotechnology Research Institute) as described previously [47]. A clear consent was obtained from each patient to publish the harvested data.

### Immunoassay for determination of camel and human IgGs reactivity against HCV peptides

ELISA plate was coated with 50 μl of HCV peptides or BSA as negative control at a concentration of 5 μg/ml, incubated for 24 h at room temperature then blocked with 100 μl of blocking buffer (2 % BSA or gelatin in PBS) for 1 h at room temperature. Then, 50 μl of polyclonal camel IgG (3 μg/ml) and IVIG (3 μg/ml) diluted in 2 % BSA or gelatin-PBS, were added in serial double-fold dilution. After 1 h of incubation at room temperature, 50 μl of diluted 1:100 mouse anti-camel IgGs and mouse anti-human IgGs [48] were added to wells containing polyclonal camel IgG and IVIG, respectively, incubated for one hour at room temperature and then 50 μl of alkaline phosphatase–conjugated anti-mouse IgG (BIO-RAD, Hercules, CA 94547, USA) diluted 1:2000 with 2 % BSA or gelatin–PBS were added. Finally, *p*-Nitrophenyl phosphate (*p*-NPP) was added for color development and absorbance recorded at 405 nm immediately.

### Cytotoxic effect of camel IgGs, α-lactalbumin, cLf, casein and intravenous immunoglobulin G (IVIGs)

Cytotoxicity effect of purified camel IgGs, α-lactalbumin, cLf, casein, or IVIGs on human separated PBMCs and Huh7.5 cells was tested by Thiazolyl Blue Tetrazolium Bromide (MTT) assay [49-51] PBMCs cells and Huh7.5 cells (10^4^) were cultured in 96-well microtiter plates in duplicate and cultured overnight at 37 °C, then the medium was refreshed with DMEM supplemented medium containing 1.0 or 2.0 mg/ml of IgGs, α-lactalbumin, cLf, casein, or IVIGs. Cells treated with proteins were incubated for 4 days at 37 °C, 5 % CO_2_ and 88 % humidity. Afterwards, 20 μl of MTT solution (5 mg MTT/ ml PBS) were added to each well. The plate was shaken at 150 rpm for 5 min, to thoroughly mix the MTT into the media. Then, it was incubated at 37 °C, 5 % CO_2_ and 88 % humidity for 5 hours to allow the MTT to be metabolized. Finally, 200 μl of dimethylsulfoxide were added to each well, placed on the shaking table at 150 rpm for 5 min and the optical density was read at 595 nm [49-51].

### Inhibition potential of camel IgGs, α-lactalbumin, cLf, casein, and IVIGs on HCV

Firstly, the interaction of IgGs, α-lactalbumin, cLf, casein, or IVIGs with HCV particles was examined by adding 1 ml of 2 % HCV infected serum (6.4 × 10^6^ copies/mL, genotype 4a) with IgGs, *α-*lactalbumin*,* cLf**,** casein, or IVIGs for 1 h at 4 °C (final concentrations of 0.5 and 1.0 mg/ml for each protein), then the mixture of HCV and protein was added to PBMCs leukocytes or Huh7.5 cells cultured as described above, and incubated for 90 min at 37 °C. The cells were washed three times with PBS and cultured for 7 days at suitable conditions, followed by washing three times with PBS and total RNA was extracted from cells. Secondly, the interaction of camel IgGs, *α-*lactalbumin*,* cLf**,** casein, or IVIGs with the human PBMCs and Huh7.5 cells (10^5^), was examined by adding these proteins at the final concentration of 1.0 mg/ml to the cells (in DMEM supplemented medium) and incubated for 1 h at 37 °C. All proteins were removed by washing the cells three times with PBS. After addition of 1 ml of medium containing 2 % infected serum (6.4 × 10^6^ copies/mL, genotype 4a) to each well, the cells were incubated for 90 min at 37 °C. The cells were washed three times with PBS and cultured for seven days at 37 °C, followed by total RNA extraction.

### HCV replication inhibition potential of camel Lf, casein, IgGs, α-lactalbumin and human IVIGs

Human PBMCs and Huh7.5 cells were washed twice in DMEM media. The PBMCs and Huh7.5 cells were suspended at 1.0 × 10^5^ cells/ml in DMEM culture media. The cells were left to adhere on the 24-well plates overnight at 37 °C, then infected with HCV-infected serum (6.4 × 10^6^ copies) in DMEM media and incubated for two days at 37 °C. Camel Lf, casein, IgGs, *α-*lactalbumin or IVIGs were added at concentrations of 0.25, 0.5, 0.75, 1.0 and 1.25 mg/ml to infected cells. Positive (infected cells with HCV) and negative control (healthy cells) cultures were included. After four days of incubation at 37 °C, total RNA was extracted from cultured cells. Second dose of cLf at concentrations of 0.25 and 0.5 mg/ml were added to the cells and incubated for another four days at 37 °C, followed by total RNA extraction.

### RNA extraction from PBMCs, Huh7.5 cells and RT-nested-PCR of HCV RNA

Extraction of RNA from PBMCs and Huh7.5 cells was performed as previously described in detail [29]. The reverse transcription-nested PCR was carried out by using plus and minus strand primers [30,43], with some modification, since the amplification process included a Rulc plasmid as internal control. The final DNA amplicon was subjected to 3 % agarose gel electrophoresis and ethidium bromide was used to visualize 174 bp for HCV and 374 bp of Rulc.

### Statistical analysis

Most measurements were repeated three times and the results are presented as the mean plus standard deviation. Data were analysed by using Student’s t-test.

## Abbreviations

HCV, Hepatitis C virus; IVIG, Intravenous immunoglobulin G; IgG, Native polyclonal immunoglobulins G; PBMCs, Peripheral blood monocyte cells; HCAbs, Heavy-chain antibodies; RT-PCR, Reverse transcriptase-polymerase chain reaction; ELISA, Enzyme linked-immunosorbent assay; BSA, Bovine serum albumin; PBS, Phosphate buffer saline; *p*-NPP, *p*-Nitrophenyl phosphate; MTT, Thiazolyl blue tetrazolium bromide; DMEM, Dulbecco’s modefied eagle medium; HAMLET, Human α-lactalbumin made lethal to tumor cells; HCIGIV, Intravenous HCV positive immunoglobulin-G; CDR3, Complementary determining region.

## Competing interest

Authors declare that they have no competing interest.

## Authors’ contributions

EME, perform tissue culture, viral screening research; NA made protein purifications and all immunoassays; EME and NA wrote the draft of manuscript; BMH, help in manuscript revision; LS, help in data organization, data analysis, manuscript revision; NAR, contributed new reagents/analytical tools and contributed in MS writing; and EMR, design research, data management, manuscript finalizing in its final form. All the authors read and approved the final manuscript.
